# Non-Destructive Detection of Internal Quality of Sanhua Plum Based on Multi-Source Information Fusion

**DOI:** 10.3390/foods15020371

**Published:** 2026-01-20

**Authors:** Weihao Zheng, Sai Xu, Xin Liang, Huazhong Lu, Pingzhi Wu

**Affiliations:** 1 College of Engineering, South China Agricultural University, Guangzhou 510642, China; 2 Institute of Facility Agriculture, Guangdong Academy of Agricultural Sciences, Guangzhou 510640, China; 3 Guangdong Academy of Agricultural Sciences, Guangzhou 510640, China; 4 Institute of Fruit Tree Research, Guangdong Academy of Agricultural Sciences, Guangzhou 510640, China

**Keywords:** Vis/NIR spectroscopy, visual image, multi-sensor data fusion, non-destructive testing, soluble solids content

## Abstract

This research addresses the limitations of traditional assembly line equipment, which is costly and impractical for narrow terrains, as well as the challenges of portable devices in large-scale detection. We propose a non-destructive testing method for assessing the internal quality of Sanhua Plums using a free-fall approach that integrates near-infrared spectroscopy and images. Through analysis of models created from spectral data collected under optimal conditions (motor speed: 6.6 r/min, integration time: 14 ms, spot diameter: 20 mm), we processed near-infrared data from 120 plums. The spectral data underwent preprocessing with polynomial smoothing (SG) and Standard Normal Variate (SNV) calibration, followed by feature extraction using Competitive Adaptive Reweighted Sampling (CARS), resulting in a prediction model for soluble solid content with R^2^ of 0.8374 and RMSE of 0.5014. Simultaneously, a prediction model based solely on visual image data achieved an R^2^ of 0.3341 and RMSE of 1.0115. We developed a multi-source information fusion model that incorporated Z-score normalization, linear weighted fusion, and Partial Least Squares Regression (PLSR), resulting in an R^2^ of 0.8871 and RMSE of 0.4141 for the test set. This model outperformed individual spectroscopy and visual models, supporting the development of an automated non-destructive system for evaluating Sanhua Plum’s internal quality.

## 1. Introduction

The iterative development of sensing, spectroscopy, and algorithms is advancing non-destructive detection [1] of internal quality of agricultural products from the laboratory to the field and production line. Near-infrared (NIR) spectroscopy [2], electronic noses [3], and machine vision [4] can instantly capture soluble solid content, acidity, and defect information without damaging the fruit, making them the core of modern grading equipment [5]. Near-infrared spectroscopy indirectly measures the chemical components of fruit via their spectral features, and this approach enhances the accuracy of measurement, thus providing clearer information on soluble solid content [6].

Currently, the application of near-infrared spectroscopy technology in online detection of soluble solid content (SSC) primarily follows two directions: “inline” [7] and “portable” [8]. However, traditional inline equipment [9] requires long belts and turntables, along with large-power motors, frequency converters, and multi-stage reduction mechanisms, which results in a large footprint and imposes strict requirements regarding foundation flatness and three-phase power supply. The complexity of the mechanical structure leads to high construction costs. Furthermore, in hilly orchards [10] where plots are scattered and roads are narrow, it is difficult to install large equipment, and small processing plants often have to forgo automated grading due to limited plant area and power capacity [11]. In contrast, while portable equipment [12] can operate independently, it relies on manual placement of each fruit, resulting in low average detection efficiency [13]. Additionally, the force of contact between the probe and the fruit surface can vary due to human factors, further exacerbating precision drift. Although machine vision [14] technology can achieve millisecond-level external grading, it has notable deficiencies when applied solely to internal quality detection: this technology can only analyze surface RGB or grayscale information and lacks direct response to the chemical composition of the fruit flesh; the correlation between skin color and soluble solid content is weak, leading to high misjudgment rates when surface defects and intrinsic quality do not align. Additionally, factors such as dust, water film, scratches, or changes in lighting can cause feature drift, forcing the system to undergo frequent recalibration [15]. The reliance on single image data for fruit quality models can lead to significant fluctuations in prediction errors, which severely hinders the development of industrial applications.

The Sanhua plum is renowned as the “king of summer fruits in Lingnan” due to its large size, small pit, crispy flesh, and sweet juice, coupled with health benefits such as appetite stimulation, heat relief, and fat reduction. However, traditional methods for detecting soluble solid content often involve fruit dissection, which is inefficient and results in high losses, making it challenging for farmers to accurately determine the optimal harvest period. Processing enterprises are also unable to achieve large-scale quality grading [16], leading to poor product consistency. Previous studies have demonstrated non-destructive quality assessment of plums using multimodal and machine learning–based approaches, including machine learning models that predict fresh fruit weight from smartphone-derived fruit dimensions with high accuracy, and deep learning–based fusion of color images and near-infrared spectral data to grade plum quality with significantly improved performance compared to single-modality methods [17,18]. Meanwhile, sellers struggle to implement premium pricing strategies for quality fruit due to information delays. Therefore, it is crucial to develop a non-destructive, intelligent, and rapid internal quality detection method for Sanhua plums. The method of gravity-driven detection can synchronize the collection of near-infrared spectral data and visual images during the free fall of the fruit, significantly overcoming the limitations of traditional detection methods. This approach not only drastically reduces the requirements for power supply, simplifying the deployment of electrical infrastructure, but also makes installation and operation in remote areas, such as hilly orchards, more convenient. Simultaneously, the compact design of the gravity drop detection system allows it to occupy a small footprint, making it well-suited for space-constrained environments, thereby facilitating quality testing for farmers and processing enterprises. Moreover, this system supports the simultaneous collection of spectral data and visual images within the same device, forming a multi-source information prediction model. This capability offers significantly higher detection precision compared to portable devices, greatly enhancing the accuracy of soluble solid content predictions.

To this end, this paper proposes and establishes a free-fall multi-source information fusion detection platform. This platform uses gravity to enable controlled free fall, synchronously capturing visible/NIR spectra and high-definition visual images within a closed dark box. The research is centered around three key issues: (1) employing a servo motor to control the rotation of a turntable, combined with photoelectric sensors to rapidly collect spectral data and visual images in real-time, ensuring that the fruit falls in a natural manner, effectively enhancing data collection efficiency and accuracy; (2) determining the optimal parameters for the platform by adjusting integration time, motor speed, light source intensity, and different collection postures, thus ensuring that optimal signal collection is achieved under various detection environments and improving overall data quality; (3) establishing a multi-source information fusion model that accurately predicts the soluble solid content of Sanhua plums by integrating spectral information and visual image data. This fusion method effectively combines the two data types, leveraging their respective advantages to improve detection accuracy and reliability. This research not only addresses the inherent limitations of traditional machine vision but also provides the Sanhua plum industry with an efficient, accurate, and cost-effective new paradigm for quality control. Its broad application potential extends to other types of spherical fruits. The design and implementation of this method make significant contributions to non-destructive and intelligent evaluations of the internal quality of Sanhua plums, effectively resolving the low efficiency and high losses associated with traditional testing methods, and providing valuable support for farmers, processing enterprises, and merchants in quality control and pricing strategies.

## 2. Materials and Methods

### 2.1. Sanhua Plum Samples

In this experiment, 190 Sanhua Plum samples were procured from the Changban Market in Guangzhou, China, with an average weight of 58.2 g. Among these samples, 70 were designated for parameter optimization experiments, while 120 were utilized for model construction and validation. All samples were immediately processed at the Guangdong Academy of Agricultural Sciences after purchase.

### 2.2. Detection Platform and Data Acquisition

#### 2.2.1. Spectral and Visual Imaging Detection Platform

**Figure 1:**
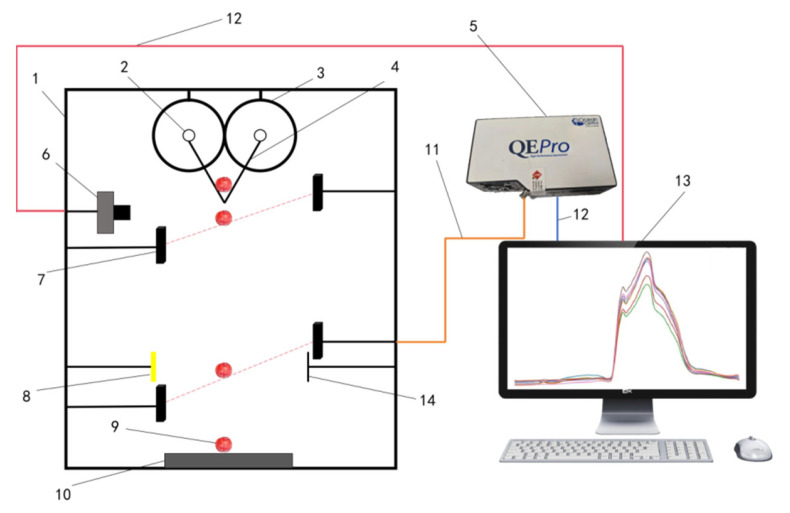
Self-Constructed Non-Destructive Internal Quality Inspection Platform for Sanhua Plums. 1. Black box. 2. Coupling (Motor control). 3. Disc. 4. Baffle. 5. Spectrometer. 6. Industrial camera. 7. Photoelectric switch sensor. 8. Light source. 9. Sanhua Plum samples. 10. Foam pad. 11. Optical fiber. 12. Data cable. 13. Computer. 14. Receiver.

The free-fall disc collection platform for Sanhua Plum is illustrated in Figure 1. During sampling, the plums were placed horizontally between two adjustable baffles. The motor drove the disc to rotate, allowing the baffles to open momentarily and initiating the free fall of the fruit. Two sets of photoelectric switches were positioned along the falling path: the first set activated an industrial camera (MER2-160-249U3M-L-HS, Daheng Imaging, Beijing, China) to capture high-definition images in real time, while the second set triggered a spectrometer (QE pro, Ocean Optics, Orlando, FL, USA, covering a range of 400–1100 nm) to concurrently record visible/near-infrared spectra. The entire process was carried out in a closed dark box to prevent ambient light interference. Adjustable parameters included light source brightness, motor speed, and the distance between the light source, sample, and receiving optical fiber, ensuring stable and repeatable imaging and spectral data collection for Sanhua Plums of varying sizes.

#### 2.2.2. Visible/Near-Infrared Spectra Collection

This study collected spectral data from 190 Sanhua Plum samples in two phases. In the first phase, 70 samples were randomly selected and placed in a temperature-controlled environment set at 25 °C and relative humidity of 45%. The free-fall disc platform was employed for parameter optimization by varying motor speeds to 6.6, 13.3, and 20 r/min, integration times to 8, 11, and 14 ms, and using spot diameters of 20, 35, 50 mm by employing three lenses of different focal lengths to assess the effect of each combination on spectral signals. The light source was turned off upon achieving stable equipment operation, and the fiber optic port was covered to collect dark current. Reference spectra were obtained using a standard white board, followed by recording sample spectra with the light source activated. Each sample was repeated three times under identical conditions, with the average taken as the final spectrum for that sample. In the second phase, based on the optimal parameters determined, spectra for the remaining 120 samples were collected again, maintaining a fixed platform position and performing three repeated measurements for each fruit. This resulted in 120 average spectra used independently for model building and validation, ensuring no overlap between data for parameter optimization and model evaluation.

#### 2.2.3. Visual Image Capture

Following the second phase protocol, visual images of the aforementioned 120 Sanhua Plum samples were captured individually using the disc collection device for model building and validation. In the Galaxy View software V2.5, the industrial camera’s white balance was set to 2.242, while the exposure time was fixed at 1000 ms, and the rising edge trigger mode was enabled. Samples were triggered for immediate capture by the photoelectric sensor, with the resulting high-definition images stored directly on a computer to ensure temporal and sequential correspondence with the spectral data.

### 2.3. Measurement of Physicochemical Values

After collecting the spectral and visual data for the free-fall Sanhua Plums, each sample was bisected along the central axis. The pulp from each half was selectively extracted and juiced using a manual juicer, with the resultant juice poured into disposable cups for identification. Subsequently, a sugar refractometer (model PAL-BX/ACID1, Atago Co., Ltd., Tokyo, Japan) underwent zero calibration with distilled water. Juices from the samples were then carefully introduced into the sensing groove of the refractometer to accurately document the soluble solid content, providing pivotal data support for subsequent research.

### 2.4. Data Analysis and Modeling

#### 2.4.1. Data Preprocessing Methods

To mitigate noise fluctuations present in the 400–1100 nm spectral data, correct scattering noise, and eliminate spectral differences originating from varying levels of scattering during measurement, five preprocessing methods were applied to the spectral data of the 120 Sanhua Plum samples used for modeling and validation: Savitzky-Golay (SG) [19], Standard Normal Variate (SNV) [20], Multiplicative Scatter Correction (MSC) [21] as well as their combinations (SG + SNV, SG + MSC) [22]. These preprocessing methods effectively enhanced the quality and stability of the spectral data while improving accuracy in subsequent modeling processes. To extract the most effective wavelengths, three feature selection methods were employed for comparative analysis: Competitive Adaptive Reweighted Sampling (CARS) [23], Successive Projections Algorithm (SPA) [24], and Uninformative Variable Elimination (UVE) [25]. The CARS algorithm assesses the importance of variables based on regression coefficients, amalgamating adaptive reweighted sampling with an exponential decay function, thus prioritizing features with high absolute regression coefficients in Partial Least Squares Regression (PLSR). CARS identified 51 feature wavelengths as optimal variables. The SPA method manages to identify redundant variables, ultimately reducing multicollinearity and selecting 61 feature wavelengths. UVE aims to eliminate uninformative variables according to their statistical significance, evaluating each variable’s relevance against randomly generated noise variables. Variables deemed uninformative based on low importance values below a predetermined threshold were discarded. UVE identified 69 feature wavelengths. The most effective one was selected for optimizing model performance by comparing the three feature extraction methods.

The visual image information was processed using both RGB and CIELab color spaces. For each image, the mean and standard deviation were calculated for the three RGB channels (R, G, B) and three Lab channels (L*, a*, b*), yielding 12 color statistical features. Additionally, texture features were extracted using the Gray Level Co-occurrence Matrix (GLCM) method [26], focusing on four properties: contrast, dissimilarity, energy, and homogeneity. These texture features were computed at four orientations (0°, 45°, 90°, 135°) with a one-pixel offset, then averaged per property to generate 4 texture features. The combined 16-dimensional visual feature vector (6 statistical features from the RGB channels, 6 statistical features from the CIELAB (L*, a*, b*) channels, and 4 GLCM texture features.) was used to characterize both color distribution and texture patterns for model optimization.

#### 2.4.2. Model Building Method

**Figure 2:**
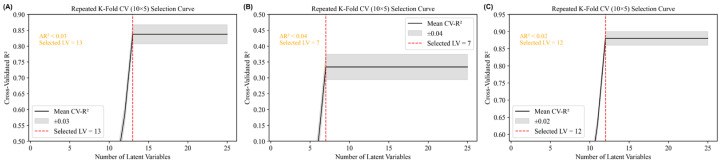
K-Fold Cross-Validation Plot. (**A**) spectral datasets. (**B**) visual datasets. (**C**) fused datasets.

**Figure 3:**
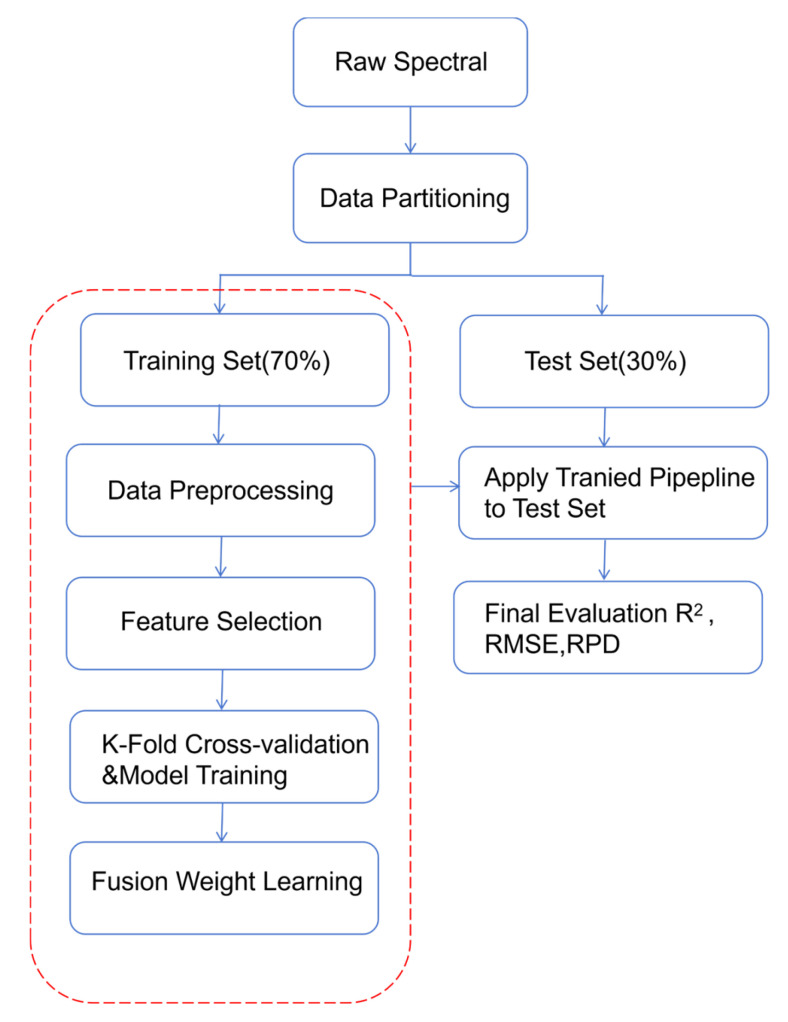
Model Building Flowchart.

The extracted spectral and visual features were randomly divided into training sets and test sets at a ratio of 7:3, with a Partial Least Squares Regression (PLSR) model established [27]. PLSR is a linear latent-variable regression method, it forms latent components (linear combinations of predictors) and then fits a linear regression model in that reduced space. Model performance was evaluated using the coefficient of determination (R^2^) and root mean square error (RMSE). The number of latent variables was determined by 5-fold cross-validation (random seed 42) with an upper bound of 25, yielding values of 13, 7, and 12 for spectral, visual, and fused datasets, respectively (Figure 2). All preprocessing, feature-selection (CARS, SPA, UVE), fusion-weight learning, and model-training steps were conducted solely on the training set. The test set was used exclusively for the final performance assessment and was never accessed during model development or selection (Figure 3).

#### 2.4.3. Multi-Source Information Fusion Method

The extracted spectral and visual image features were first standardized using Z-score normalization to eliminate dimensional disparities. The normalized features were then fused by applying linear regression to calculate the fusion weights for both spectral and visual features. These weighted features were concatenated to form a unified matrix. Finally, the fused features were input into a Partial Least Squares Regression (PLSR) model to predict soluble solid content with high precision.

### 2.5. Statistical Analysis

Statistical analysis was performed to evaluate the performance and robustness of the predictive models. Model accuracy was assessed using the coefficient of determination (R^2^) and root mean square error (RMSE). The number of latent variables in the Partial Least Squares Regression (PLSR) model was determined using 5-fold cross-validation, with optimal values selected based on minimizing prediction errors.

In addition, RPD (Ratio of Performance to Deviation) was calculated to evaluate model robustness, with higher RPD values indicating better model prediction accuracy. All statistical computations were performed using Python (v3.8) and the scikit-learn library, and results were considered statistically significant when *p*-values were less than 0.05.

## 3. Results and Discussion

### 3.1. Distribution of SSC

**Figure 4:**
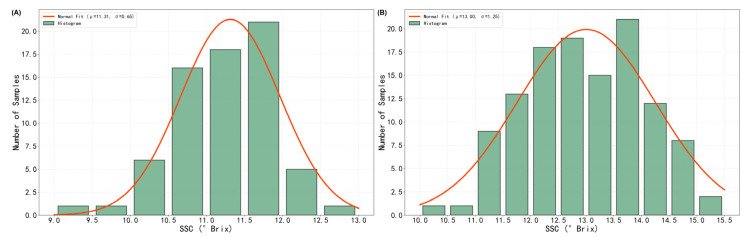
Distribution of Soluble Solid Content (SSC) in Sanhua Plums. (**A**) Histogram of SSC values for the 70 Sanhua plums used in the parameter-optimization experiment. (**B**) Histogram of SSC values for the 120 plums used to build the prediction model.

The SSC of the 70 Sanhua plums used for the parameter-optimization experiment ranged from 9.3 to 12.9 °Brix, with a normal fit of μ = 11.31 °Brix and σ = 0.65 °Brix (A). For the 120 fruits employed to build the prediction model, the interval was 10.2–15.3 °Brix, yielding μ = 13.00 °Brix and σ = 1.25 °Brix (B). These distribution characteristics indicate that the dataset used for model building spans a wider range of SSC values than the optimization set, which is beneficial for developing robust prediction models (Figure 4).

### 3.2. Visible/Near-Infrared Spectroscopy Detection Model

#### 3.2.1. Impact of Integration Time on Modeling Results

**Figure 5:**
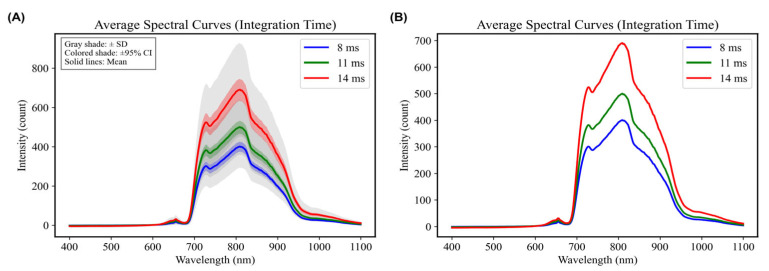
Average spectra under different Integration Times. (**A**) With associated error margins (Standard Deviation and 95% Confidence Interval). (**B**) Without error margins.

Under a fixed motor speed of 6.6 r/min and a fixed spot diameter of 20 mm, visible/near-infrared spectra were collected from Sanhua Plums in free fall by varying integration times (8 ms, 11 ms, and 14 ms). Notably, integration times exceeding 15 ms exhibited significant spectral disorder, prompting the comparison of 8 ms, 11 ms, and 14 ms (Figure 5). Analyzing the average spectral graphs for the 70 Sanhua Plum samples across various integration times revealed consistent peak and valley positions, with prominent peaks observed at approximately 720 nm and 820 nm, along with a distinctive valley around 740 nm [28]. Over the entire 400–1100 nm wavelength range, an increase in integration time resulted in an overall rise in spectral intensity [29]. This suggests that longer integration times enable the spectrometer to capture more effective internal information from the plums. A PLSR model was constructed using spectral data collected from the 70 samples under the aforementioned integration time settings to explore the influence of integration time on modeling outcomes further. Modeling results indicated that the optimal performance occurred at an integration time of 14 ms: the coefficient of determination for the training set (R^2^c) reached 0.9171, accompanied by a root mean square error (RMSEC) of 0.1888; for the test set, R^2^v attained 0.8265, with RMSEV recorded at 0.2728, demonstrating that extending integration time substantially enhances prediction accuracy and model robustness (Table 1). This improvement at 14 ms likely reflects a stronger spectral signal relative to noise, as longer integration allows greater photon accumulation from internal fruit constituents, thereby aiding the regression model in capturing relevant chemical information associated with SSC.

#### 3.2.2. Impact of Motor Speed on Modeling Results

**Figure 6:**
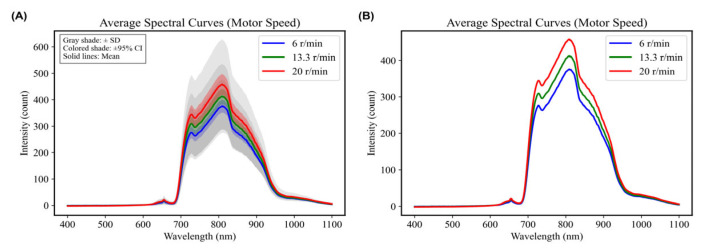
Average spectra at different Motor Speeds. (**A**) With associated error margins (Standard Deviation and 95% Confidence Interval). (**B**) Without error margins.

Under fixed conditions of 11 ms integration time and 20 mm spot diameter, visible/near-infrared spectra were collected from Sanhua Plums in free fall while varying the motor speed (6.6 r/min, 13.3 r/min, and 20 r/min). Evidence of spectral disorder emerged at motor speeds exceeding 20 r/min, prompting the establishment of this speed as the upper limit. Comparison of average spectral graphs obtained from the 70 samples at different motor speeds revealed consistent peak and valley positions across speeds. As motor speed increased, the integration area also expanded, leading to increases in overall captured average spectral intensity (Figure 6). Further analysis using PLSR modeling revealed that the model provided the best performance at a motor speed of 6.6 r/min: R^2^c for the training set reached 0.8790, with RMSEC at 0.2416; for the test set, R^2^v attained 0.7114, while RMSEV was recorded at 0.3309. An increase in motor speed led to diminished model performance, potentially due to the expanded integration area requiring improved efficiency in spectral data collection, thus increasing susceptibility to noise and scattering interference in captured spectra (Table 2). This trend suggests that lower motor speeds help maintain spectral signal stability by reducing motion-induced variation, which in turn supports better model calibration and prediction.

#### 3.2.3. Impact of Spot Size on Modeling Results

**Figure 7:**
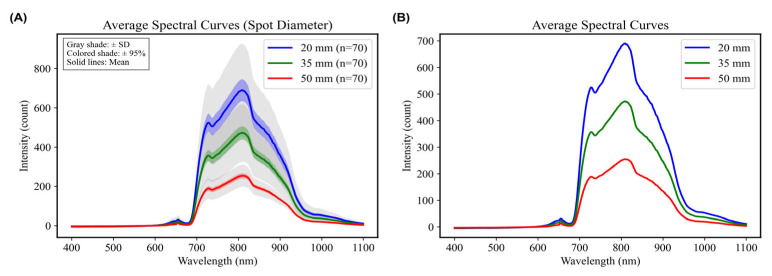
Average spectra at different Spot Diameter. (**A**) With associated error margins (Standard Deviation and 95% Confidence Interval). (**B**) Without error margins.

Under a fixed integration time of 14 ms and a motor speed of 6.6 r/min, spectra of Sanhua Plums were collected using a large spot size (diameter 50 mm), a middle spot size (diameter 35 mm), and a small spot size (diameter 20 mm) [30]. The acquired average spectra demonstrated that, compared to the variations in integration times, the spot size exerted a more pronounced influence on spectral morphology and amplitude. While peak and valley wavelengths for both spot sizes were consistent, at 720 nm and 820 nm, the smaller spot size exhibited significantly higher intensity (Figure 7). This phenomenon may be attributed to the stronger focusing ability of the smaller spot size, which concentrates beam energy and minimizes losses via shorter penetration paths through the plum’s skin and flesh, ultimately allowing the spectrometer to collect more effective information reflective of internal quality. Given the heterogeneous internal structure of the Sanhua Plum, the reduction of the spot area enhanced the density of local information covered by the beam, leading to richer signal detail. Further modeling using PLSR on spectral data obtained under varying spot size conditions revealed that modeling performance with the small spot size was superior: R^2^c for the training set reached 0.9171, with RMSEC at 0.1888; R^2^v for the test set attained 0.8265, with RMSEV measured at 0.2728 (Table 3). These results indicate that a smaller illumination spot can improve the collection of informative spectral features, likely by reducing background averaging and focusing more on localized internal compositional differences relevant to SSC prediction.

#### 3.2.4. Influence of Fall Posture on Modeling Results

**Figure 8:**
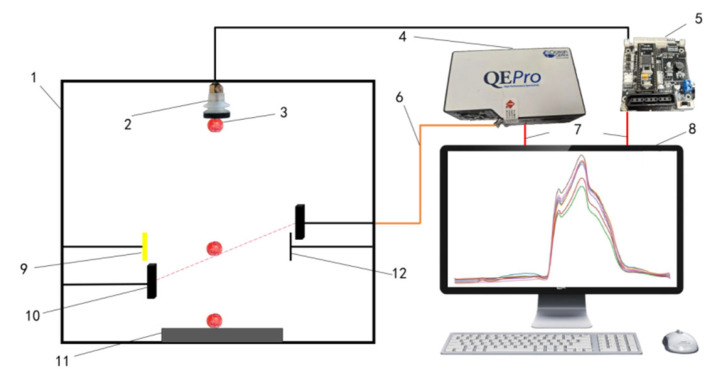
Schematic diagram of suction cup device for air pump. 1. Black box. 2. Vacuum suction cup. 3. Sanhua Plum samples. 4. Spectrometer. 5. Microcontroller. 6. Optical fiber. 7. Data cable. 8. Computer. 9. Ligh.t 10. Photoelectric switch sensor. 11. Foam pad. 12. Receiver.

**Figure 9:**
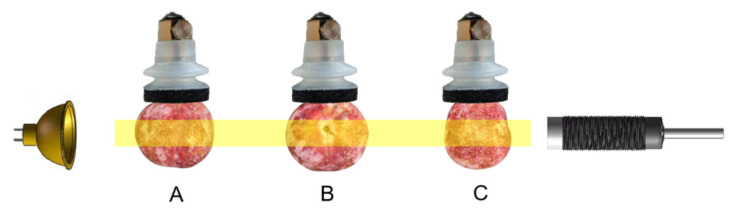
Schematic diagram of three different postures. (**A**) The light source illuminates the equatorial side (with the fruit stem facing the suction cup side). (**B**) The light source illuminates the equatorial side (with the equatorial side facing the suction cup side). (**C**) Light source shining on the top side of the fruit (equatorial side facing the suction cup side).

The baffle release of Sanhua Plums during collection could impact the posture of the fruit. A free-fall collection platform was designed using suction cups to further investigate the influence of posture on predictive modeling during free fall (Figure 8). The microcontroller controlled the air valve, allowing the pump to release air and enabling the plums to fall in varied postures. As the plums passed through the photoelectric sensor, the spectrometer collected real-time visible/near-infrared (Vis/NIR) spectral information from the plums during free fall in different postures (Figure 9).

**Figure 10:**
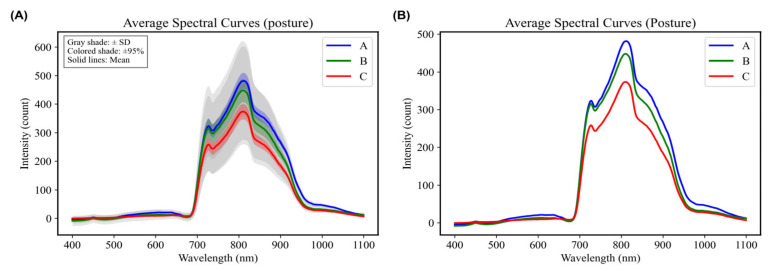
Average spectra under different Postures. (**A**) With associated error margins (Standard Deviation and 95% Confidence Interval). (**B**) Without error margins.

Comparative analysis of average spectra for the plums in different postures indicated similar spectral waveforms with notable peaks at 720 nm and 820 nm (Figure 10). Differences between postures A and B were minimal, while posture C exhibited lower spectral intensity compared to the other two postures. This observation likely stems from postures A and B illuminating the equatorial surface, while posture C illuminated the stem end, which requires longer light pathways, thus capturing more spectral energy [31]. PLSR modeling demonstrated that the model formed from posture A, which illuminated the equatorial surface, yielded optimal results: R^2^c and RMSEC for the training set were recorded at 0.8457 and 0.3892, respectively. In contrast, R^2^v and RMSEV for the test set reached 0.8027 and 0.4399. This is attributed to the more comprehensive internal quality information obtained from illuminating the equatorial surface (Table 4). This suggests that fruit orientation affecting the illuminated surface can influence the richness of spectral information acquired, and that equatorial illumination captures more representative internal quality features for SSC prediction under free-fall conditions.

#### 3.2.5. Establishment of the Visible/Near-Infrared Spectroscopy Detection Model

**Figure 11:**
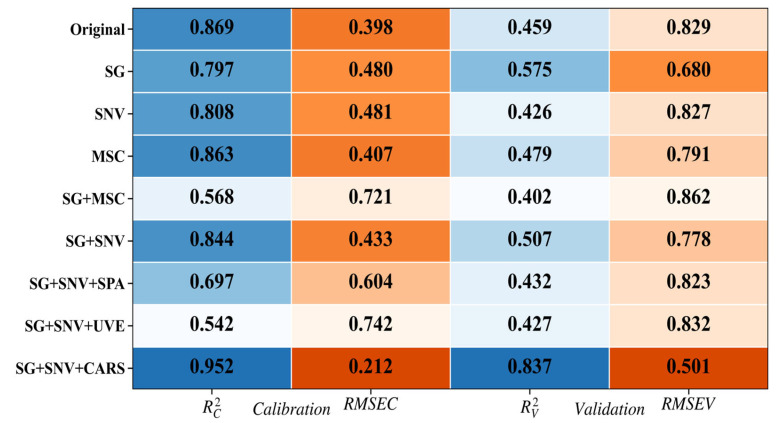
Model Results of Different Preprocessing and Feature Extraction Algorithms Processing. (Blue color intensity corresponds to higher R^2^, orange color intensity corresponds to lower RMSE).

To enhance the performance of the free-fall Sanhua Plum model, efforts were made to reduce speed and extend integration times without compromising detection rates while using light sources with the smallest achievable spot size for data collection to augment model performance. Using the free-fall disc collection platform, the motor speed was adjusted to 6.6 r/min, with a 20 mm-diameter light source and an integration time of 14 ms, positioning the plums in posture A for spectral and image data collection. A random sampling strategy was employed to partition the 120 Sanhua Plum samples into training and prediction sets. To mitigate interference, six preprocessing methods were applied: Savitzky-Golay (SG) smoothing, Standard Normal Variate (SNV), Multiplicative Scatter Correction (MSC), and their respective combinations (SG + SNV, SG + MSC). To identify the most relevant feature wavelengths from the preprocessed spectral data, three feature selection methods were employed: Competitive Adaptive Reweighted Sampling (CARS), Successive Projections Algorithm (SPA), and Uninformative Variable Elimination (UVE) [32]. The experimental results indicate that leveraging SG smoothing accompanied by SNV calibration for preprocessing, with CARS for feature selection and PLSR modeling, yielded superior accuracy in predicting soluble solid content for Sanhua Plum. The detection results displayed R^2^c and RMSEC for the training set of 0.9530 and 0.2090, respectively, and R^2^v and RMSEV for the test set of 0.8389 and 0.4976 (Figure 11). These results demonstrate that the combined preprocessing and variable selection strategy effectively enhances model robustness and prediction performance, likely by reducing noise and focusing the model on the most informative spectral features.

### 3.3. Visual Image Detection Model

**Figure 12:**
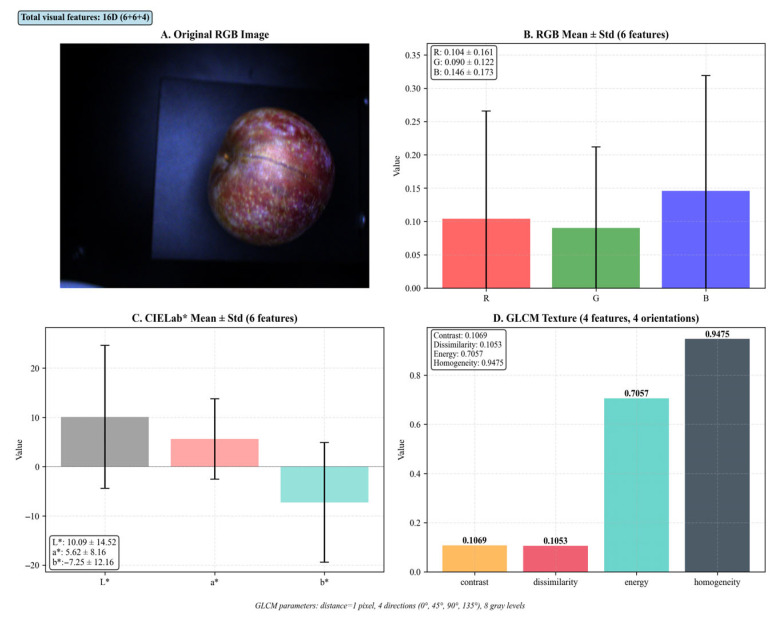
Visual feature extraction from plum fruit images.

Visual images of the plums were analyzed using both RGB and CIELab color spaces [33]. A 16-dimensional visual feature vector was constructed by concatenating: (1) the mean and standard deviation of the three RGB channels (6 features), (2) the mean and standard deviation of the three Lab* channels (6 features), and (3) four Haralick texture descriptors—contrast, dissimilarity, energy and homogeneity—extracted from the gray-level co-occurrence matrix (GLCM) at four orientations (0°, 45°, 90°, 135°) with a one-pixel offset and averaged per property (4 features) (Figure 12). This joint color–texture signature was mean-centered and variance-scaled before modelling. The modeling outcomes indicated that the prediction model based on these 16 visual features exhibited coefficients of determination for the training and validation sets of 0.7197 and 0.3341, respectively, suggesting a moderate correlation. The visual features of Sanhua Plum contain some internal quality information conducive to predicting soluble solid content.

### 3.4. Multi-Source Information Fusion Model

**Figure 13:**
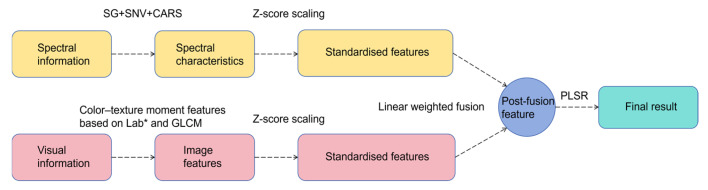
Flowchart of Feature Fusion Between Spectral Information and Image Information.

**Figure 14:**
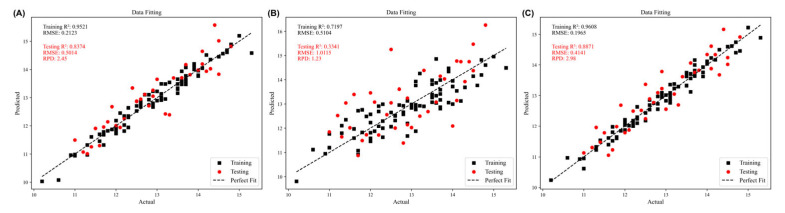
PLSR detection for different datasets. (**A**) PLSR detection processed by SG + SNV + CARS. (**B**) PLSR detection of soluble solid content based on grayscale features of images. (**C**) PLSR detection based on multi-source information fusion.

While visual image features exhibit some correlation with the internal soluble solid content of Sanhua Plum, their predictive accuracy remains limited. To improve precision, a lightweight data-fusion model was proposed that integrates VIS/NIR spectral features (after preprocessing and CARS extraction) and visual features (15-dimensional CIE-Lab* color–texture moments) through a simple linear weighting strategy (Figure 13). First, each feature block was subjected to Z-score standardization [34], forcing zero mean and unit variance across every channel and thereby eliminating dimensional disparities between spectral and image information. The standardized vectors were concatenated and fed to an ordinary-least-squares regression that automatically learned the optimal linear weights minimizing the SSC prediction error; spectral and visual coefficients were then used to re-scale the respective features before fusion. This weighted stack, which simultaneously emphasizes SSC-relevant spectral absorption bands and discriminative colour–texture patterns, was finally regressed against SSC using PLSR (Figure 14). The approach mitigates the limitations of single-modality models without additional complexity. The fusion model achieved R^2^c = 0.9608 and RMSEC = 0.1965 for the training set, and R^2^v = 0.8871, RMSEV = 0.4141, RPD = 2.98 for the test set. RPD exceeds the common threshold of 2.5, indicating quantitative prediction capability. Compared with spectral-only PLSR, test-set R^2^ improved by 0.0497 and RMSEV decreased by 0.0873; relative to the vision-only model, R^2^ rose by 0.553 and RMSEV fell by 0.5974 (Table 5). These results demonstrate that the linear-fusion scheme significantly enhances SSC prediction accuracy.

### 3.5. Discussion

Multi-source and data fusion strategies have been widely explored to enhance fruit quality prediction beyond single-modality approaches. For instance, in cherry tomatoes, data-layer fusion of visible/near-infrared (Vis/NIR) and NIR spectral imaging combined with machine learning achieved determination coefficients (R^2^) greater than 0.9 for both soluble solid content (SSC) and titratable acidity (TA), outperforming individual spectral models and highlighting the benefit of leveraging complementary spectral bands for multiple quality attributes [35]. In oranges, a machine vision–Vis/NIR fusion framework using a colour-correction 1D-CNN significantly reduced RMSEP by 36.4% compared to partial least squares regression, demonstrating that integrating visual color cues with spectral data can mitigate spectral distortion from color variation and improve SSC prediction [36].

Similarly, mid-level data fusion of Vis/NIR hyperspectral imaging and FT-NIR spectroscopy for yellow-flesh kiwifruit resulted in marked enhancements in SSC prediction (test R_P_^2^ ≈ 0.914) and reductions in RMSEP relative to models based on individual spectral sources, indicating that combining information from different spectral domains can strengthen model robustness [37]. In apples, low-level and mid-level fusion strategies integrating hyperspectral imaging (HSI) and Vis/NIR data achieved improved SSC prediction performance (R_p_^2^ ≈ 0.927) compared to single spectral methods, underscoring the value of data fusion for capturing complex biochemical signatures in heterogeneous fruit tissues [38].

Beyond purely spectral fusion, other multi-sensor fusion studies have also shown advantages. For example, combining four nondestructive sensing techniques (including Vis-SWNIR spectroscopy and spectral scattering) for apple quality assessment demonstrated that fused systems were more effective than individual sensors for predicting SSC and firmness, suggesting that integrating orthogonal sensing modalities can exploit synergistic information [39]. Research on mandarin SSC prediction has additionally shown that including physical properties such as fruit diameter and color alongside Vis/NIR spectral features further improved model accuracy compared to models based solely on spectral data, illustrating the positive impact of incorporating external factors into predictive models [40].

In comparison, the free-fall multi-source fusion model developed in this study for Sanhua plum SSC achieved a test-set R^2^v of 0.8871 and an RPD of 2.98. While absolute R^2^ values are modestly lower than some hyperspectral fusion benchmarks, our model demonstrates competitive quantitative prediction with a simpler and more compact acquisition setup that synchronously collects Vis/NIR spectral data and visual colour–texture features in a single device configuration. The linear weighting fusion strategy used here avoids the high computational cost and large training datasets often required by deep learning methods, yet still captures complementary internal chemical and external morphological cues that single-modality models lack. Importantly, the relatively lightweight design and real-time free-fall acquisition mechanism mean that this model maintains robust performance without the need for multiple spectral ranges, bulky imaging hardware, or complex calibration procedures, which are common in other fusion studies. This suggests that even with a more modest data volume and hardware configuration, effective multi-source fusion can be achieved when modalities are thoughtfully combined, balancing prediction performance with practical deployability for online fruit quality assessment.

## 4. Conclusions

Although the proposed method shows promising SSC prediction performance, some limitations should be noted. The sample size (n = 120) is relatively small, which may affect model generalizability across seasons and orchards. The free-fall acquisition introduces motion and orientation variability, potentially impacting spectral and image consistency. Additionally, this study focused only on soluble solid content; other quality traits such as firmness and acidity warrant future investigation.

This study successfully designed a free-fall-based detection device for assessing the internal quality of Sanhua Plum. A comparative analysis of motor speed, integration time, spot size, and collection posture was conducted, identifying optimal parameters that do not compromise detection efficiency. Specifically, a motor speed of 10 r/min, an integration time of 14 ms, a small spot diameter of 20 mm, and illumination directed at the equatorial surface yielded optimal modeling results. A multi-source fusion information model was established based on these parameters, combining spectral information and visual image data. The visible/near-infrared (Vis/NIR) spectroscopy effectively captures the chemical changes associated with variations in soluble solid content, while visual technology records the physical changes in external features resulting from these differences. The performance of this model exhibited significant improvement compared to models constructed solely with spectral data or exclusively with visual image data. This method demonstrates a strong predictive capability for the soluble solid content in Sanhua Plum samples, with rapid detection speeds along with non-destructive testing during the process, making it well-suited for real-time online monitoring. Therefore, the proposed non-destructive detection method based on free-fall multi-source information fusion illustrates high feasibility, providing a solid research foundation and reference for the internal quality assessment of similar spherical fruits.

## Figures and Tables

**Table 1:** Detection accuracy of the PLSR model at different Integration Times for 70 Sanhua Plum samples used in the optimization experiment.

Spot Diameter	Motor Speed	Integration Time	Train Set		Test Set	
			$R^{2}$	RMSE	$R^{2}$	RMSE
20 mm	6.6 r/min	8 ms	0.7732	0.3258	0.6806	0.3370
20 mm	6.6 r/min	11 ms	0.8935	0.1764	0.7149	0.3185
20 mm	6.6 r/min	14 ms	0.9171	0.1888	0.8265	0.2728

**Table 2:** Detection accuracy of the PLSR model at different Motor Speeds for 70 Sanhua Plum samples used in the optimization experiment.

Spot Diameter	Motor Speed	Integration Time	Train Set		Test Set	
			$R^{2}$	RMSE	$R^{2}$	RMSE
20 mm	6.6 r/min	11 ms	0.8935	0.1764	0.7149	0.3185
20 mm	13.3 r/min	11 ms	0.8090	0.2865	0.6755	0.3732
20 mm	20 r/min	11 ms	0.7836	0.2803	0.6284	0.4255

**Table 3:** Detection accuracy of the PLSR model at different Spot Diameter for 70 Sanhua Plum samples used in the optimization experiment.

Spot Diameter	Motor Speed	Integration Time	Train Set		Test Set	
			$R^{2}$	RMSE	$R^{2}$	RMSE
20 mm	6.6 r/min	14 ms	0.9171	0.1888	0.8265	0.2728
35 mm	6.6 r/min	14 ms	0.8582	0.2387	0.7561	0.3104
50 mm	6.6 r/min	14 ms	0.8297	0.2705	0.6972	0.3605

**Table 4:** Detection accuracy of the PLSR model under different Postures for 70 Sanhua Plum samples used in the optimization experiment.

Posture	Train Set		Test Set	
	$R^{2}$	RMSE	$R^{2}$	RMSE
A	0.8457	0.3892	0.8027	0.4399
B	0.8010	0.4419	0.7254	0.5190
C	0.7188	0.5253	0.6974	0.5449

**Table 5:** Detection accuracy of models using different collected information for 120 Sanhua Plum samples.

Collect Information	Train Set		Test Set		
	$R^{2}$	RMSE	$R^{2}$	RMSE	RPD
spectral information	0.9521	0.2121	0.8374	0.5014	2.45
image information	0.7197	0.5104	0.3341	1.0115	1.23
Multi source information	0.9608	0.1965	0.8871	0.4141	2.98

## Data Availability

The original contributions presented in the study are included in the article. Further inquiries can be directed to the corresponding author.

## References

[1] Guo M., Wang K., Lin H., Wang L., Cao L., Sui J. (2024). Spectral data fusion in nondestructive detection of food products: Strategies, recent applications, and future perspectives. Compr. Rev. Food Sci. Food Saf..

[2] Lee A., Shim J., Kim B., Lee H., Lim J. (2022). Non-destructive prediction of soluble solid contents in Fuji apples using visible near-infrared spectroscopy and various statistical methods. J. Food Eng..

[3] Khorramifar A., Sharabiani V.R., Karami H., Kisalaei A., Lozano J., Rusinek R., Gancarz M. (2022). Investigating changes in pH and soluble solids content of potato during the storage by electronic nose and Vis/NIR spectroscopy. Foods.

[4] Palumbo M., Cefola M., Pace B., Attolico G., Colelli G. (2023). Computer vision system based on conventional imaging for non-destructively evaluating quality attributes in fresh and packaged fruit and vegetables. Postharvest Biol. Technol..

[5] Anjali, Jena A., Bamola A., Mishra S., Jain I., Pathak N., Sharma N., Joshi N., Pandey R., Kaparwal S. (2024). State-of-the-art non-destructive approaches for maturity index determination in fruits and vegetables: Principles, applications, and future directions. Food Prod. Process. Nutr..

[6] Ahmed Z.F., Abdalla A.K., Kaur N., Wu F. (2025). Insights into recent developments and obstacles in automated fruit ripeness classification. Green Technol. Sustain..

[7] Waqar M., Memon A.M., Sabih M., Alhems L.M. (2024). Composite pipelines: Analyzing defects and advancements in non-destructive testing techniques. Eng. Fail. Anal..

[8] Wang D., Ding C., Feng Z., Ji S., Cui D. (2023). Recent advances in portable devices for fruit firmness assessment. Crit. Rev. Food Sci. Nutr..

[9] Rajmohan S., Mendem M.T., Vanam S.S., Thalapally P.K. Online grading of fruits using deep learning models and computer vision. Proceedings of the 4th International Conference on Information Management & Machine Intelligence.

[10] Li Z., Li C., Zeng Y., Mai C., Jiang R., Li J. (2024). Design and Realization of an Orchard Operation-Aid Platform: Based on Planting Patterns and Topography. Agriculture.

[11] Liu T., Yang J., Yang Z., Duan Y. (2022). Techno-economic feasibility of solar power plants considering PV/CSP with electrical/thermal energy storage system. Energy Convers. Manag..

[12] Liu L., Zareef M., Wang Z., Li H., Chen Q., Ouyang Q. (2023). Monitoring chlorophyll changes during Tencha processing using portable near-infrared spectroscopy. Food Chem..

[13] Fulgêncio A., Resende G.A.P., Teixeira M.C.F., Botelho B.G., Sena M.M. (2022). Screening method for the rapid detection of diethylene glycol in beer based on chemometrics and portable near-infrared spectroscopy. Food Chem..

[14] Yu K., Zhong M., Zhu W., Rashid A., Han R., Virk M.S., Duan K., Zhao Y., Ren X. (2025). Advances in computer vision and spectroscopy techniques for non-destructive quality assessment of citrus fruits: A comprehensive review. Foods.

[15] Kaur N., Somasundram C., Razali Z., Ahmed Z.F.R. (2024). Sustainable Aloe vera/chitosan-based edible coatings reduce postharvest loss of stored fresh figs (*Ficus carica* L.). Front. Sustain. Food Syst..

[16] Wang H., Mei M., Li J. (2023). Research progress on non-destructive detection of internal quality of fruits with large size and thick peel: A review. Agriculture.

[17] Sabouri A., Bakhshipour A., Poorsalehi M., Abouzari A. (2025). Machine learning techniques for non-destructive estimation of plum fruit weight. Sci. Rep..

[18] Liu X., Tong W., Di B., Zhang L., Lin J. (2025). Non-Destructive Detection and Grading of Plum Quality Based on Multimodal Data. Sensors.

[19] Shawky E., Nahar L., Nassief S.M., Sarker S.D., Ibrahim R.S. (2024). Spice authentication by near-infrared spectroscopy: Current advances, limitations, and future perspectives. Trends Food Sci. Technol..

[20] Shin S.K., Lee S.J., Park J.H. (2025). Prediction of Soil Properties Using Vis-NIR Spectroscopy Combined with Machine Learning: A Review. Sensors.

[21] Guo P., Li T., Gao H., Chen X., Cui Y., Huang Y. (2021). Evaluating calibration and spectral variable selection methods for predicting three soil nutrients using Vis-NIR spectroscopy. Remote Sens..

[22] Li H., Zhu L., Li N., Liu Z., Wang L., Chitrakar B., Xu D., Cui Z., Tang Y., Hu L. (2024). NIR spectroscopy for quality assessment and shelf-life prediction of kiwifruit. Postharvest Biol. Technol..

[23] Li L., Hu D.-Y., Tang T.-Y., Tang Y.-L. (2023). Non-destructive detection of the quality attributes of fruits by visible-near infrared spectroscopy. J. Food Meas. Charact..

[24] Liu J., Sun J., Wang Y., Liu X., Zhang Y., Fu H. (2025). Non-Destructive Detection of Fruit Quality: Technologies, Applications and Prospects. Foods.

[25] Han Q.-L., Lu J.-F., Zhu J.-J., Lin L., Zheng Z., Jiang S.-T. (2025). Non-destructive detection of freshness in crayfish (*Procambarus clarkii*) based on near-infrared spectroscopy combined with deep learning. Food Control.

[26] Hao Y., Zhang L., Qiao S., Bai Y., Cheng R., Xue H., Hou Y., Zhang W., Zhang G. (2022). Breast cancer histopathological images classification based on deep semantic features and gray level co-occurrence matrix. PLoS ONE.

[27] Ji F., Li F., Hao D., Shiklomanov A.N., Yang X., Townsend P.A., Dashti H., Nakaji T., Kovach K.R., Liu H. (2024). Unveiling the transferability of PLSR models for leaf trait estimation: Lessons from a comprehensive analysis with a novel global dataset. New Phytol..

[28] Xiao B., Li S., Dou S., He H., Fu B., Zhang T., Sun W., Yang Y., Xiong Y., Shi J. (2024). Comparison of leaf chlorophyll content retrieval performance of citrus using FOD and CWT methods with field-based full-spectrum hyperspectral reflectance data. Comput. Electron. Agric..

[29] Hu J., Chen G.J., Xue C., Liang P., Xiang Y., Zhang C., Chi X., Liu G., Ye Y., Cui D. (2024). RSPSSL: A novel high-fidelity Raman spectral preprocessing scheme to enhance biomedical applications and chemical resolution visualization. Light Sci. Appl..

[30] Polasi P.K., Vellela S.S., Narayana J.L., Simon J., Kapileswar N., Prabu R.T., Rashed A.N.Z. (2024). Data rates transmission, operation performance speed and figure of merit signature for various quadurature light sources under spectral and thermal effects. J. Opt..

[31] Liu P., Zheng Y., Tian H., Xu H., Xie L. (2024). Enhancing fruit SSC detection accuracy via a light attenuation theory-based correction method to mitigate measurement orientation variability. Food Res. Int..

[32] Xiao Z., Tian H., Zhuo C., Zhao K., Yu Y., Mu Q., Xue X. (2025). A rapid calibration method for the discrete element model of straw fodder particle mixtures based on the UVE-PLS-GD algorithm. Biosyst. Eng..

[33] Marefat S., Shayanfar A., Monajjemzadeh F. (2025). Developments in image-based colorimetric analysis methods and applications of CIElab color space in pharmaceutical sciences: A narrative review. Int. J. Pharm. X.

[34] Liu X., Tan B., Xiang P., Huang W., Zhang X. (2025). Multimodal Data Fusion Based Intelligent Grading System for Kiwifruit Maturity Using Spectral-Impedance Properties. J. Food Process Eng..

[35] Tan F., Mo X., Ruan S., Yan T., Xing P., Gao P., Xu W., Ye W., Li Y., Gao X. (2023). Combining vis-NIR and NIR spectral imaging techniques with data fusion for rapid and nondestructive multi-quality detection of cherry tomatoes. Foods.

[36] Sun Z., Tian H., Hu D., Yang J., Xie L., Xu H., Ying Y. (2025). Integrating deep learning and data fusion for enhanced oranges soluble solids content prediction using machine vision and Vis/NIR spectroscopy. Food Chem..

[37] Cevoli C., Iaccheri E., Fabbri A., Ragni L. (2024). Data fusion of FT-NIR spectroscopy and Vis/NIR hyperspectral imaging to predict quality parameters of yellow flesh “Jintao” kiwifruit. Biosyst. Eng..

[38] Lin Y., Fan R., Wu Y., Zhan C., Qing R., Li K., Kang Z. (2025). Combining hyperspectral imaging technology and visible-near infrared spectroscopy with a data fusion strategy for the detection of soluble solids content in apples. J. Food Compos. Anal..

[39] Guo Z., Chen X., Zhang Y., Sun C., Jayan H., Majeed U., Watson N.J., Zou X. (2024). Dynamic nondestructive detection models of apple quality in critical harvest period based on near-infrared spectroscopy and intelligent algorithms. Foods.

[40] Huang C., Cai J., Zhou Y., El-Seedi H.R., Guo Z. (2022). Fusion models for detection of soluble solids content in mandarin by Vis/NIR transmission spectroscopy combined external factors. Infrared Phys. Technol..

